# Sex selection from Islamic point of view

**Published:** 2014-04

**Authors:** Lotfollah Dezhkam, Hakime Dezhkam, Iman Dezhkam

**Affiliations:** 1 *Department of Maaref, Jahrom University of Medical Sciences, Jahrom, Iran.*; 2 *Young Researchers Club, Islamic Azad University, Jahrom Branch, Jahrom, Iran.*; 3 *Student Research Committee, Jahrom University of Medical Sciences, Jahrom, Iran.*

**Keywords:** *Islam*, *Sex selection*, *PGD*, *Ericsson method*, *Pre implantation*, *Genetic diagnosis*

**Dear Editor**

Now there are three ways to select the sex of the fetus before implantation (1, 2). Two of these methods are scientifically proven that are used in the fertility and infertility centers (3). Two types of pre implantation methods (PGD and Ericsson method) used for social sex selection. Both of them are based on actively rendering the second sex chromosome to be either a Y chromosome (resulting in a male), or an X chromosome (resulting in a female). Another way to determine the sex of an embryo before implantation is called PGD (pre implantation genetic diagnosis). In this procedure, after ovarian stimulation multiple oocytes are removed from the mother. The eggs are fertilized in the laboratory using the father's sperm in a technique called in vitro fertilization (IVF). As the embryos develop through cleavage, a blastomere is removed from each embryo and then assessed for the presence of Y and X chromosome and separated by sexual chromosome. Embryos of the desired gender are transferred back in the mother's uterus (4).

There are two ways to select the sperm sex; Micro Sort and Ericsson method (5, 6). In this way the sperm that contain the desired sex chromosome can be selected and used for fertilization of female gametes. In Ericsson as sperm passes through albumin gradient, the differences in mass between the X and Y chromosomes cause the females dragged down by the weight of the extra "leg" of the X sex chromosome. The method has a 70-72% success rate for boys and a 69-75% success rate for girls (2). The third method is the determination of sperm sex chromosomes in sperm sorting by flow cytometry. Sperm sorting is an advanced technique that sorts sperm "in vitro" by flow cytometry. This shines a laser at the sperm to distinguish X and Y chromosomes and can automatically separate the sperm out into different samples. The technology is already in commercial use for animal farming (7). It is currently being trialed on humans in the US under the trademark Micro Sort; it claims a 90% success rate but is still considered experimental by the FDA (3). 

There are also methods for sex selection after implantation which can be performed by prenatal sex discernment, followed by sex-selective abortion of any offspring of the unwanted sex that is unaccepted in Islam and there is not the place to discuss (8). There are great ethical debates about being true or false using these methods in sex selecting in American and European ethics committees. Primary care physicians questioned whether women could truly express free choice under pressure from family and community. Other concern is that sex selection led to invasive medical interventions in the absence of therapeutic indications that could result in child neglect of the lesser-desired sex (9). We want to answer this concern in Islamic view and show whether it is true or false to use this methods in Islam. Currently the sex selection is done for two medical and non-medical reasons that in medical reason preventing genetic disease to occur in a child and Non-medical reason is done merely for the sake of parental interest and desire to have a son or daughter.

From the Islamic point of view (Shia jurisprudence), there is no barrier for using the two methods accepted by the FDA and the reasons cited by the Ethics Committee of the American and European to ban the use of these methods is just for the possibility and fear of future risk that are talked about and those reasons are not notable at all and we can simply make strategies to prevent them. The only thing that limit the use of Micro Sort and Ericsson method, is arrangements of doing (Unlawful see and Unlawful touch) (10, 11). 

With regard to the above, we could conclude, first the use of these two methods (PGD and Ericsson method) is allowed for medical items and second for non-medical items must exist a necessity or hardship to allow to do (12). This task is dependent on the organizations and institutions review ethical and medical considerations and ambiguities to set a framework for using these methods.
